# Smoking cessation intervention prior to orthopedic surgery: A study protocol to determine patient outcomes and feasibility

**DOI:** 10.18332/tpc/152608

**Published:** 2022-08-29

**Authors:** Kalle Kainiemi, Antti Malmivaara, Sari Sillman-Tetri, Mervi Lasander, Minna Heinonen, Tellervo Korhonen, Juhani Sand, Tiina Laatikainen, Antti Kyrö

**Affiliations:** 1 Department of Acute Medicine, Päijät-Häme Central Hospital, Lahti, Finland; 2 Institute of Public Health and Clinical Nutrition, University of Eastern Finland, Kuopio, Finland; 3 Department of Public Health and Welfare, National Institute for Health and Welfare, Helsinki, Finland; 4 Research Unit, Orton Orthopedic Hospital, Helsinki, Finland; 5 Surgical Outpatient Department, Päijät-Häme Central Hospital, Lahti, Finland; 6 Department of Health Security, National Institute for Health and Welfare, Helsinki, Finland; 7 Faculty of Medicine, Department of Nursing Science, University of Turku, Turku, Finland; 8 Institute for Molecular Medicine Finland FIMM, University of Helsinki, Helsinki, Finland; 9 Department of Administration, Tampere University Hospital, Tampere, Finland

**Keywords:** complications, surgery, smoking, hand surgery, orthopedics, smoking cessation intervention

## Abstract

The aim of the study is to analyze the effect of smoking and smoking cessation on the incidence of complications among orthopedic and hand surgery patients, and to determine the feasibility of smoking cessation intervention, as well as factors predicting success in smoking cessation. Orthopedic and hand surgery patients will be invited to participate in the study, which will recruit 550 participants (at least 20% daily smokers). A participant will be defined as a daily smoker if he/she reports daily smoking and/or laboratory tests show active smoking. Data will be collected using a self-reported questionnaire and from medical records. Smokers will receive information about the benefits of smoking cessation and will be encouraged to quit. Medication or nicotine replacement therapy will be prescribed. Laboratory tests will be taken two weeks before and two weeks after surgery. Follow-up phone calls will be made at 3, 6, and 12 months after surgery. The primary outcome is any complication, defined as a prolonged stay in hospital or any additional visit to or measure taken by a health service during the 12 months after surgery. Data on complications are mainly obtained from personal health records and from the information received at the follow-up; the rest of the data will be collected from the register of healthcare-associated infections. Secondary outcomes are the number and types of complications. The sample (n=550) was calculated to observe a 10% difference in complications between smokers and non-smokers (5% alpha level and 80% power), considering a 10% drop-out rate. Logistic regression and log-linear models will be used for data analyses.

## INTRODUCTION

Smoking has many adverse effects on orthopedic and hand surgery patients. It impairs blood circulation in the area of surgical intervention^1,2^, oxygenation of tissues^3,4^, and immune response to infections^5,6^. Smoking increases the risk of thrombosis in vessels^7,8^. Further, it impairs the function of osteoblasts^9^, decreases the resorption of calcium from the intestines^10^, and lowers the level of vitamin D^5^.

Smoking also hinders wound healing^11^. Tibial, femoral, humeral, clavicular, and wrist navicular fractures heal more slowly in smokers than in non-smokers^12-17^. The healing of ankle arthrodesis^18^, high tibial osteotomy^19^, and ulnar shortening osteotomy^20^, is also slower and more uncertain among smokers than among non-smokers. Smokers have more complications on hip and knee arthroplasties compared with non-smokers^21,22^. The outcome of spinal surgery is worse for smokers than for non-smokers^23,24^. Smokers have six-fold overall risk of surgical wound infections compared with that of non-smokers^25^.

Nåsell et al.^26^ have shown that in the smoking cessation intervention group, 20% compared with 38% in the control group had surgical complications after acute fracture surgery. In another study by Møller et al.^27^, the overall complication rate was 18% in the smoking cessation intervention group and 52% in the control group, and the median length of hospital stay was 11 and 13 days, respectively. Glassman et al.^28^ have shown that smoking cessation for longer than six months after a posterior instrumented lumbar spinal fusion reduces the rate of nonunion to 17% compared with 26% for those who continued to smoke after surgery. The nonunion rate was 14% among non-smokers. In a study of elective orthopedic and special surgery patients with a smoking cessation intervention of four weeks before and four weeks after surgery, the incidence of postoperative complications was 21% in the intervention group and 41% in the control group^29^.

Hejblum et al.^30^ have shown in their cost-benefit analysis that a smoking cessation intervention of hip and knee arthroplasty patients saved on average 117 € per patient in 2008. The average 90-day cost of total joint arthroplasty with a smoking cessation intervention was 32 US$ less than the respective cost without intervention in 2017^31^. In the US, the mean cost of a total hip arthroplasty with a periprosthetic infection was over 88 thousand US$ and that without a periprosthetic infection over 25 thousand US$, from 2007 to 2011^32^. So, the mean cost of a periprosthetic infection after a total hip arthroplasty was close to 63 thousand US$ . In all, smoking cessation preoperatively is a very valuable and cost-effective method of reducing the risks of postoperative complications, especially infections of orthopedic patients. With this intervention, a significant amount of money could be saved. Further, smoking cessation is a very effective method to improve health^33^. Tosi et al.^34^ have reported that an intervention program among patients who had a low-energy fracture and who received counselling on calcium and vitamin-D supplementation, weight-bearing exercise, smoking cessation, and fall prevention, reduced the risk of secondary fractures.

It is very challenging to motivate smokers to quit before orthopedic or hand surgery. This is even though we know very well that the risk of complications is much lower when a patient stops smoking before surgery. Lindström et al.^29^ found that a significant proportion of their patients were not interested in smoking cessation and they wanted only to concentrate on the forthcoming surgery. Bender et al.^35^ showed, in their study of patients who had surgery for long bone nonunion, that only 8% of smokers had stopped smoking at six weeks after surgery.

### Aims

This article describes the protocol of our study which aims to determine: 1) the effects of smoking and smoking cessation intervention on the incidence of complications after orthopedic surgery; 2) the feasibility of preoperative smoking cessation intervention and its effect on reducing smoking; and 3) which factors predict success in smoking cessation. The secondary aims of the study are: a) to compare the incidence of different types of complications in the whole study population and in different subgroups of orthopedic and hand surgery patients who are smokers, non-smokers or quitters who stopped smoking during the preoperative intervention; and b) to determine which other factors in addition to smoking, such as chronic diseases and medication, explain the incidence of complications.

### Hypotheses

1. Smoking patients have more complications compared with non-smoking patients and patients continuing smoking despite smoking cessation intervention have more complications than initially smoking patients who are stopping smoking because of the intervention.
2. Smoking cessation of patients with planned arthroplasties or heavy spine operations or other heavy operations, and of patients with low or moderate nicotine dependence, is successful more often than in other patients.

## METHODS

### Recruitment

**Figure 1:**
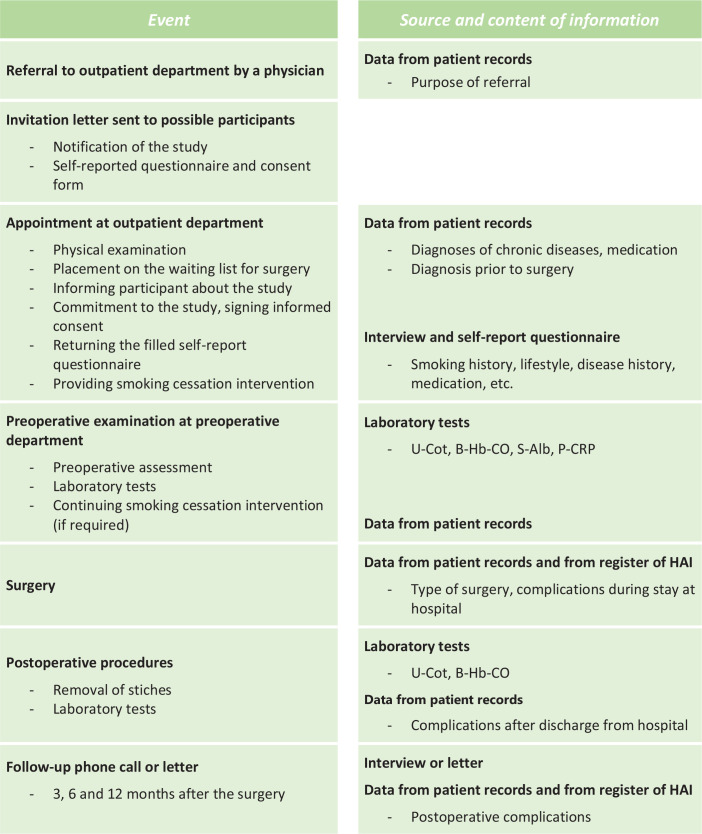
Sequence of procedures

Participants for the study will be recruited among patients visiting the orthopedic or hand surgery outpatient department and who are on the waiting list for elective orthopedic or hand surgery in Päijät-Häme Central Hospital, Lahti, Finland (Figure 1). The orthopedic surgeon (who registers the patient on a waiting list) and nurse decide if the patient is eligible to participate in the study.

The eligibility criteria are at least 18 years of age and that the planned surgery will be directed at the bone. Patients under 18 years of age, patients with an acute fracture, and patients awaiting other types of surgery are excluded from the study. A total of 550 patients will be recruited aiming to have at least 20% of them daily smokers, corresponding approximately to the current smoking prevalence in Finland^36^. The number of smokers will be followed up during the process so that more could be recruited during the recruitment period if their proportion seems too small.

### Data collection

Participants fill in a self-reported questionnaire collecting detailed background information on chronic diseases, medication, socio-economic status, physical activity, and use of alcohol. The questionnaire includes questions on present and past smoking; use of electronic cigarettes, snus, and pipe tobacco; and exposure to passive smoking. Additional and clarifying information such as diagnoses of chronic diseases and prescribed medication will be collected from participants’ personal health records. Information on the orthopedic diagnosis and planned orthopedic surgery will be filled in on the study form (Figure 1).

During the preoperative assessment at about two weeks before the surgery, laboratory tests will be taken to verify the smoking status of the participants and to differentiate the use of other nicotine products (by cotinine of urine, U-Cot and carboxyhaemoglobin, Hb-CO) and to exclude malnutrition (albumin of serum, S-Alb) and other significant catabolic processes (C-reactive protein, P-CRP) (Figure 1). Participants’ smoking history will be based on classification used in surveys conducted by the Finnish National Institute for Health and Welfare (THL) and categorized from 1 to 5 as follows: 1=daily smoker, 2=occasional smoker, 3=quit smoking 1–12 months ago, 4=quit smoking over 12 months ago, and 5=never smoker^36^.

During the intervention, participants’ smoking status will be confirmed using the following criteria. A participant is determined as a smoker if he/she reports daily smoking and/or U-Cot is above 250 µg/L and Hb-CO above 2%. A participant who reports exposure to passive smoking and/or has a U-Cot of 100–250 µg/L, is categorized as a passive smoker. Among non-smokers, U-Cot is typically <50 µg/L and Hb-CO <2%. Hb-CO is used to specify users of snus or NRT, among whom Hb-CO is <2% but U-Cot is elevated.

### Intervention

Smokers will be given verbal and written information about health and economic benefits of smoking cessation and encouraged to quit smoking. The Fagerström test for nicotine dependence (FTND) will be conducted to estimate the level of nicotine dependence of smokers^37^. If a smoker has moderate nicotine addiction, NRT will be recommended. Prescription medication to aid smoking cessation will be considered if a participant had strong nicotine dependence, i.e. smoked over 10 cigarettes per day and first cigarette was lit within 30 minutes after waking up, or a previous attempt to quit smoking yielded significant withdrawal symptoms^38^.

The smoking cessation intervention will be given in the outpatient department, by the surgeon, the nurses, or both. In discussions with smokers, the aim is to share information, persuade, motivate, and offer skills training and social support to quit smoking. The intervention will be performed during an outpatient appointment when the patient is registered on the waiting list and also during the preoperative visit. After surgery, when the stitches will be removed, Hb-CO and U-Cot tests will be repeated to verify participants’ smoking status to verify successful smoking cessation. Participants will be followed up to 12 months after surgery. Participants will receive a phone call or letter at 3, 6, and 12 months after surgery, asking them if any problems or complications had occurred (Figure 1). The description and ICD codes of complications will be recorded in the study form.

### Data from operation and definition of complications

The Finnish register of healthcare-associated infections (HAI) includes information about the operations of all surgically treated patients, regardless of whether the patient has had a postoperative infection or not. For this study, information about the surgery and any postoperative infection will be obtained from that register. A complication will be defined as any event which causes additional medical or surgical treatment, an additional radiological or laboratory test, a prolonged stay in hospital or any additional visit to a healthcare unit, following the criteria used by Nåsell et al.^26^. Data on complications have been mainly collected and will be completed from participants’ personal health records during their perioperative stay in hospital. These data will be combined with all available personal health data during 12 months after surgery and the information received during follow-up phone calls at 3, 6, and 12 months after surgery (Figure 1).

### Primary and secondary outcomes

The primary outcome is any complication occurring within 12 months after surgery. The number and types of complications (for example rapidly occurring complications, repeat surgery, and infections, stratified by types of surgery) are considered as secondary outcomes. Subgroup analysis based on patient characteristics and comorbidities will be carried out. Successful smoking cessation is a feasibility outcome.

### Sample size calculation and power analysis

The sample size was calculated assuming that the proportion of smokers in the patient population is comparable with the smoking prevalence in the Finnish adult population, i.e. about 20%^36^. The other assumption was that with appropriate support before surgery, as many as half of the smokers could quit smoking. The sample (n=550) was calculated to observe 10% difference in postoperative complications between smokers and non-smokers, with 5% alpha error and 80% power, considering a 10% drop-out rate.

### Data analyses

To analyze the risk of primary outcome, i.e. the postoperative complications among smokers, non-smokers, and quitters, a logistic regression model will be used. The effectiveness of the smoking cessation intervention will be assessed using logistic regression analyses. Associations of smoking status and background variables with complications will be analyzed using log-linear models considering patients’ gender, age, and comorbidities.

### Study ethics procedures

Participation in the study is voluntary. All eligible patients will be informed about the study and motivated to participate by an invitation letter. Those willing to participate will be given a detailed information brochure and asked to sign an informed consent form. Ethical approval for the study was received from the Regional Ethical Committee of Tampere University Hospital, Finland (Approval number: R15129; Date: 22 September 2015). Permission for the study was granted by the chief medical director of Päijät-Häme Central Hospital, Lahti, Finland.

### Patient and public involvement statement

Neither patients nor members of the public were and will be involved in the design or conduct of this study.

## DISCUSSION

In studies concerning smoking and orthopedic surgery, the definition of smoker and/or smoking status has been diverse. For example, ex-smokers are classified as non-smokers; mostly, smoking status is based only on patients’ self-report without any biochemical verification. Bender et al.^35^ showed in their study of patients who had had surgery for long bone non-union that 23% of participants reporting to be former smokers and 6% of participants reporting to be non-smokers had serum cotinine values suggesting that they were active smokers. In this study, laboratory tests will be performed to biochemically verify participants’ smoking status to increase the evidence for the effect of smoking on the outcome of orthopedic surgery. Thus, some participants who report to be non-smokers could be classified as active smokers based on the laboratory tests.

Ehnert et al.^39^ showed that the rate of complications after joint arthroplasty of smokers exceeds that of non-smokers and increasing number of cigarettes smoked raises the rate of complications. Also, they showed that bone formation and inflammatory response markers of smokers are lower than those of non-smokers. However, the aim of this study is to gather evidence on the effects of biochemically confirmed smoking and smoking cessation on the outcome of defined, but otherwise unselected orthopedic and hand surgery operations and on feasibility and effects of a preoperative smoking cessation intervention. The intervention will be carried out in real-life settings, so all smokers will receive the smoking cessation intervention following a standard protocol. Randomization to smoking cessation intervention (yes or no) among active smokers would be an unethical study design. Further aims are to assess the factors affecting the feasibility and outcome of the intervention and its effects on costly complications which are more likely to occur among smokers than non-smokers. This valuable information could be used in future to support orthopedic or hand surgery patients in smoking cessation.

### Strengths and limitations

The strengths include that the study population will be recruited from a central hospital serving the whole population of a catchment area. Further, laboratory tests will be taken to biochemically verify participants’ smoking status to increase accuracy of smoking status assessment among the patients. A potential limitation is that the smoking cessation interventions will be carried out during the clinical work in the orthopedic and hand surgery outpatient departments and surgical wards at the non-teaching central hospital. Further, because of previous evidence of effectiveness of smoking cessation intervention, randomized trial design was not feasible. Finally, there will be probably overrepresentation of non-smoking patients for the study due to the requirement of filling a long questionnaire, complying with extra laboratory tests to be taken twice, and answering follow-up phone calls three times presumed during the study.

## CONCLUSIONS

This study will provide new and valuable information on smoking cessation in reducing complications in surgery and will also demonstrate feasibility of smoking cessation intervention in a hospital treating orthopedic and hand surgery patients.

## Data Availability

The data supporting this research cannot be made available for privacy reasons.

## PROTOCOL REGISTRATION

The protocol is registered on ISRCTN registry with study ID ISRCTN16961081.
